# Immunogenicity and Antigenicity of the Ectodomain of Rabies Virus Glycoprotein Stably Expressed in HEK293T Cells

**DOI:** 10.7150/ijms.87134

**Published:** 2023-08-15

**Authors:** Qingqing Li, Renhe Yan, Na Bai, Zhenglan Tan, Qing Yu, Heng Su, Xiwen Wei, Andrew Li, Xueji Chen, Zhenyu Li, Yuezhong He, Hongwei Li, Xiangxin Li, Yingying Mao

**Affiliations:** 1 Clinical Laboratory, Affiliated Foshan Maternity & Child Healthcare Hospital, Southern Medical University, Foshan, 528000, China;; 2 School of Laboratory Medicine and Biotechnology, Southern Medical University, Guangzhou, 510515, China;; 3 Guangzhou Bioneeds Biotechnology CO., LTD, Guangzhou, 510000, China;; 4 Department of Nuclear Medicine, Yuxi People's Hospital of Yunnan Province,Yuxi, 653100, China;; 5 Department of Biomedical Engineering, The Johns Hopkins University School of Medicine, Baltimore, 21205, USA;; 6 South China Institute of Biomedicine, Guangzhou, 510000, China.

**Keywords:** Rabies virus, Envelope glycoprotein, Secretory expression, Lentiviral vector, HEK 293T

## Abstract

Rabies continues to be a huge threat to public health. The rabies virus envelope glycoprotein (RABV G) is a major rabies virus antigen and contains neutralizing epitopes, which are primary candidates for subunit vaccines and diagnostic antigens. However, the production and purification of rRABV G while retaining its antigenic and immunogenic remains to be a challenge. Here, we aimed to establish a platform for rRABV G production and purification, and determine the immunogenicity and antigenicity of rRABV G. The cDNA fragment encoding the soluble form of RABV G was synthesized and cloned into a lentiviral expressing vector. Recombinant lentiviral vector LV-CMV-RABV G-eGFP was packaged, titered, and then transduced into HEK 293T cells. The cell culture supernatant was purified using nickel affinity chromatography and subsequently confirmed through Western Blot analysis and indirect enzyme-linked immunosorbent assay (ELISA). The ELISA utilized human sera obtained from individuals who had been vaccinated with the human commercial Purified Vero Cells Rabies Vaccine (PVRV). Notably, we observed a neutralizing antibody response in immunized pigs rather than in mice. This discrepancy could potentially be attributed to factors such as the instability of the rRABV G protein, variations in host responses, and variances in the adjuvant used. Taking all these findings into account, the rRABV G protein generated in this study exhibits promise as a potential vaccine candidate for the prevention of rabies.

## Introduction

Rabies is a zoonoses caused by the Rabies virus (RABV) or *Rhabdoviridae Lyssavirus,* which is a negative-stranded RNA virus 1. Rabies causes 59,000 (95% CI: 25-159,000) human deaths per year; most of which is in Asia and Africa where dogs are major disease carriers 2. Rabies virus can be transmitted from infected animals to humans via bite wounds, scratches, or contact with mucous membranes. The case fatality rate is almost 100% after the onset of clinical symptoms. Strategies to prevent human rabies infection include wound cleaning, post-exposure vaccination and inoculation of human rabies immunoglobulin (HRIG). Conventional rabies vaccines consist of inactivated viruses with the same antigenic characteristics as wild type viruses. The whole inactivated viruses can elicit virus-neutralizing antibodies against rabies viruses resulting in protection against lethal intracerebral challenge 3, 4. Despite this, several research groups have demonstrated that traditional rabies vaccination does not provide sufficient post-exposure protection 5-7. Another challenge is ensuring that human rabies vaccines and HRIG are affordable and accessible for exposed patients. Rabies infection continues to be a potential public health issue in many countries due to poverty, ignorance, and lack of effective vaccine or lack of HRIG. Thus, it remains as a significant unmet medical need to develop a more powerful post-exposure vaccine capable of providing the benefits of both rabies vaccine and human rabies immunoglobulin to exposed patients. The risk of production and administration of the whole inactivated rabies vaccines and the multi-vaccination schedule should also be improved for a new type of RABV vaccine development 8, 9.

RABV is a single-strand RNA virus containing five proteins, nucleoprotein, phosphoprotein, matrix protein, glycoprotein, and large protein, respectively 10. RABV G is the major protective antigen responsible for eliciting protective immunity against rabies 11. The complexity of the oligomeric rabies virus glycoprotein hampers the studies on its structure and function as well as the establishment of a rRABV G subunit vaccine. Furthermore, production and purification of rRABV G while retaining its antigenic and immunogenic remains to be a challenge 12. In this study, we utilized a RABV G recombinant lentiviral vector to create a HEK 293T cell line that exhibited stable expression of RABV G at elevated levels. We thoroughly investigated the production and purification processes of this cell line. Subsequently, both mice and pigs were immunized with purified truncated RABV G, and we assessed their serum levels of specific virus neutralization antibody (RVNA) using a fluorescent antibody virus neutralization (FAVN) test 13.

## Materials and Methods

### Plasmids construction and Cell cultures

**Figure 1:**
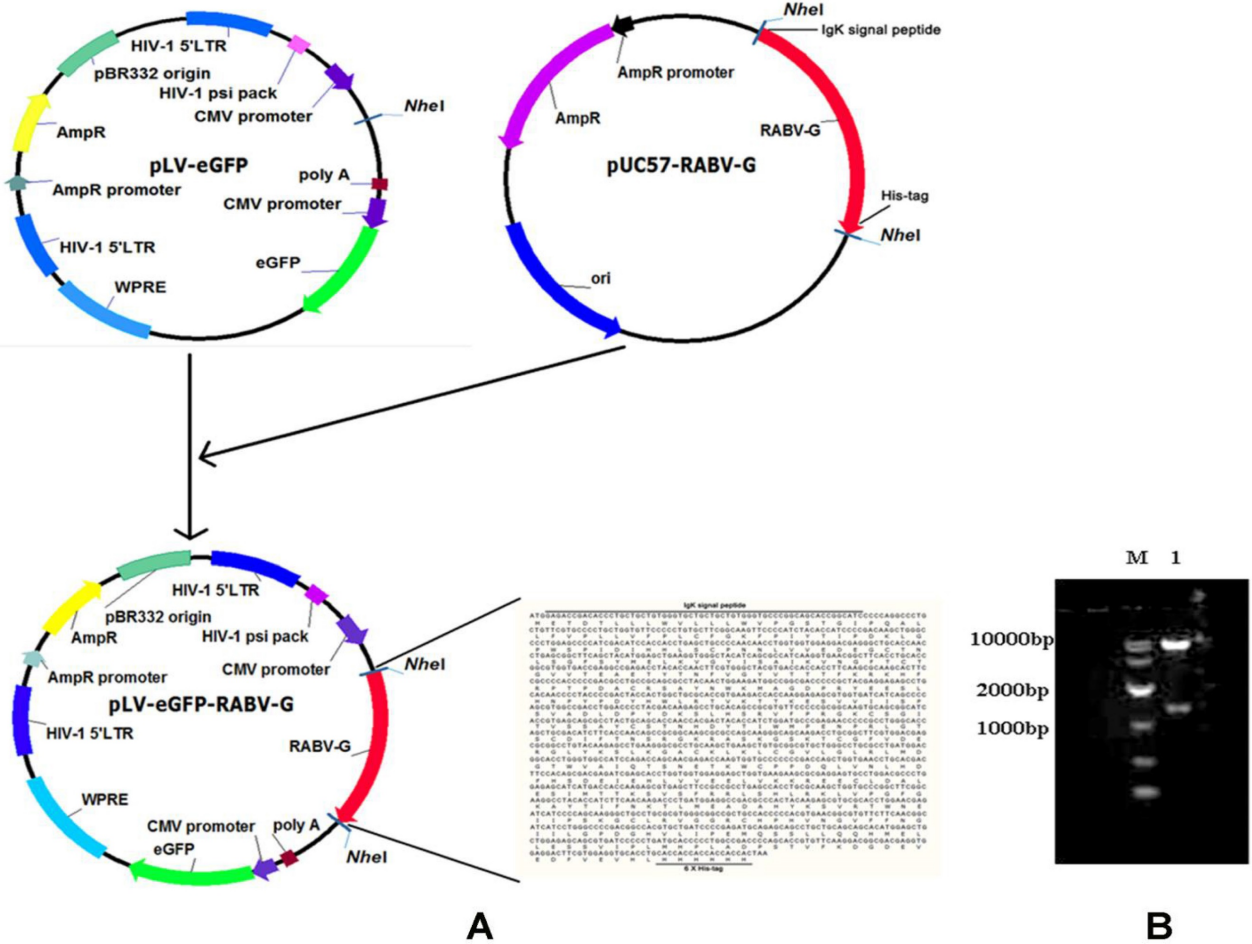
** Construction and identification of a recombinant RABV G lentiviral vector pLV-eGFP-RABV G.** (A) Diagram of the constructed recombinant plasmid pLV-eGFP-RABV G. (B) Identification of the recombinant plasmid pLV-eGFP-RABV G via digestion with *Nhe* I enzyme. Two DNA fragments were observed with expected sizes.

A pLV-eGFP plasmid containing a CMV promoter and the packaging plasmids, pMD2.G and psPAX2, were stored in our laboratory 14. Human embryonic kidney (HEK) 293T cells (ATCC, Manassas, VA, Catalogue #: CRL-3216) were cultured following the ATCC cell culture guide. The CVS-11 RABV standard strain was provided by Changchun Veterinary Research Institute. The codon-optimized cDNA fragment encoding the soluble form of RABV G is characterized by: deletions of the C-terminal hydrophobic anchor (transmembrane) and cytoplasmic domains of the glycoprotein, fusion with a polyhistidine tag, and replacement of the N terminal viral signal peptide with the Ig kappa chain signal peptide. This was synthesized and cloned into a lentiviral expression plasmid pLV-eGFP (Figure 1). The sequence of the cDNA fragment is derived from the rabies virus strain CTN-1V5G (Accession number: JN234418). The resulting plasmid was designated as pLV-eGFP-RABV G, which was validated by restriction enzyme digestion and partial sequencing.

### Generation of lentiviral vectors

A three-plasmid packaging system was used to package the lentiviral vector as described previously 14. Briefly, 5×10^6^ HEK 293T cells were seeded in a 10 cm dish and later transfected at 90% confluency. A transfection solution mixture was made by: 1.25 µg of shuttle plasmid pMD2.G, 3.75 µg of packaging plasmid psPAX2, 5 µg of transfer expression plasmid DNA, and 125 µl of 2 mM CaCl_2_ in 500 µl of deionized distilled water; CaCl_2_/DNA was then added dropwise, while vortexing, to an equal volume of 2× HBS, for total of 1 ml. This mixture was added to the 10 cm dish and the cells were then maintained in 5% CO_2_ at 37℃. The packaged recombinant lentiviruses were harvested from the supernatant of the cell cultures at 48 h post transfection and quantified in copies/ml by real-time PCR.

### Cell transduction *in vitro* and detection of GFP expression

293T cells were prepared in a 24-wells plate and transduced with lentiviral vectors as described previously 14. Briefly, on the following day, we transduced them with the packaged recombinant lentivirus at a MOI of 1000, and cell culture media were replaced with fresh DMEM containing 10% FBS and maintained for 3 days at 37℃ and 5% CO_ 2_ at 6 h post transduction.

### Establishment of RABV G-expressing clonal cell lines

The transduced cells were clonally expanded by limiting dilution as described previously 15. Briefly, clones in good condition were picked out two to three weeks later, then, the cells were cultured and passaged once every three days at a ratio of 1:5. The fluorescence from the eGFP was examined under an Olympus Model BX41 fluorescent microscope (Olympus, Tokyo, Japan) and mean fluorescence intensities were measured by a FACS Calibur flow cytometer (Becton Dickinson, MA, USA). The transduced cells were also evaluated for RABV G expression by Western Blot using anti-His monoclonal antibody.

### Purification of RABV G from cell culture supernatant

The recombinant RABV G (rRABV G) was purified through nickel column affinity chromatography as previously described 16. Briefly, about 200ml culture supernatant were collected and filtered using 0.45 μM syringe filters. After mixing with a binding buffer (20 mM sodium phosphate, 0.5 M NaCl, 40 mM imidazole, pH 7.4), the supernatants were loaded onto nickel affinity columns (GE Healthcare, US). The columns were washed with washing buffer (50 mM NaH2PO4, 300 mM NaCl, 20 mM imidazole) to remove unbound proteins. Before eluting His-tagged RAVB-G, we first removed nonspecific proteins with buffer containing 20 mM sodium phosphate, 0.5 M NaCl, and 100 ~ 200 mM continuous concentration of imidazole, PH 7.4. Then, rRABV G was eluted using 20 mM sodium phosphate, 0.5 M NaCl, 500 mM concentration of imidazole, PH 7.4. The purity was confirmed by 10% SDS-PAGE and Western Blot analysis. The purified rRABV G concentration was determined by BCA Protein Assay Kit (Thermo, USA).

### SDS-PAGE and Western Blot analysis

SDS-PAGE and Western Blots were performed as described previously. Anti-β-actin monoclonal antibody was purchased from Cell Signaling Technology. Anti-His monoclonal antibody and the horseradish peroxidase-conjugated (HRP) goat anti-mouse IgG were purchased from Sangon Biotech (Shanghai, China). The neutralizing anti-RABV G protein mAbs S053 and S037 were kindly provided by Dr Lin from Southern Medical University 17, 18. RABV positive serum samples were obtained from patients vaccinated with commercial human Vero-cell rabies vaccine. The serum level of antibody against RABV was detected using a fluorescent antibody virus neutralization (FAVN) test at the OIE Reference Laboratory for Rabies in Changchun, China. HRP-conjugated goat anti-human IgG (HRP) was purchased from Abcam (USA).

### Stability analysis of rRABV G protein

In this experiment, rRABV G protein was diluted in PBS buffer to achieve a final concentration of 100 μg/ml. Two different stabilizers, namely 50 mM Glycine or Trehalose, were used. The samples were aliquoted and stored at either 4℃ or -80℃ for temporary (3 days) or long-term (30 days) preservation, respectively. To assess the stability of the rRABV G protein, two analytical techniques were employed: SDS-PAGE/Western Blot and ELISA using the S053 monoclonal antibody as described below.

### Indirect ELISA

ELISA was performed to identify rRABV G antigenic property. Briefly, ELISA plates (Costar®, Corning, US) were coated with 100 μl of 0.05 M carbonate bicarbonate coating buffer (pH 9.6) containing either purified rRABV G (100 ng/well) or control protein Zika virus NS1 expressed in 293T cells developed at our lab by incubating for 16 h at 4℃. To reduce non-specific binding, 10% skim-milk was used to block the plates (100 μl/well). Serum samples diluted to 1:100 in PBS containing 10% skim-milk (BIO-RAD, USA) were added in triplicate to microtiter plates (100 μl/well) and incubated at 37℃ for 1 h. The plates were then washed five times in PBST and incubated with HRP-conjugated goat anti-human antibodies (Abcam, USA) (diluted to 1:60,000 in PBST) for 1 h at 37℃. The plates were then washed five times with PBST. Tetramethylbenzidine substrate (TMB) solution (Genstar, Shanghai) was added to each well (100 μl/well) and incubated for 10 min at 37℃. The reaction was stopped with 50 μl of 2 M sulfuric acid (H_2_SO_4_) per well. The plates were read at 450 nm with microplate reader (Bio-Rad, USA).

The sera of vaccinated samples were from persons vaccinated with commercial human Vero-cell Rabies vaccine and the levels of antibodies against RABV were measured using FAVN test at the OIE Reference Laboratory for Rabies in Changchun, China. The sera of non-vaccinated sample were collected from uninoculated healthy persons. Human serum samples come from Nanfang Hospital (Guangzhou, Guangdong, China). Written informed consent was obtained from each patient, and the research protocols were approved by the Ethics Committee of Nanfang Hospital.

### Fluorescent antibody virus neutralization test

Viral neutralizing antibody (VNA) titers of immunized animals against RABV were determined by fluorescent antibody virus neutralization (FAVN) test as described previously. Briefly, 3-fold serial dilutions of standard serum (0.5 IU/ml) and test serum samples were prepared in quadruplicate in a multi-well plate and mixed with 50 μl of CVS-11. After incubation at 37℃ in a humidified 5% CO_2_ incubator for 1 hour, a 50 μl suspension containing 2 × 10^4^ BHK-21 cells was added and incubated for 48 hours. The cells were fixed at 4℃ by treatment with 80% acetone for 30 min and stained with FITC-labeled mouse anti-RABV-N monoclonal antibodies (Veterinary Research Institute, Changchun, China). Fluorescence was observed by UV microscopy (Olympus), and the RVNA titers were calculated using the Spearman-Karber formula. Antibody titers were expressed in International Units per milliliter (IU/ml) using standard serum as a reference.

### Mice immunization with purified rRABV G

Four groups (each of six animals) of 4-6 weeks old BALB/c mice were maintained in the Laboratory Animal Center, Southern Medical University, Guangzhou, China. Animal ethics approval for the use of laboratory animal was obtained from the Animal Care and Use Committee (ACUC) of Southern Medical University. Experimental mice were inoculated intramuscularly with 1 μg or 5 μg of Montanide™ ISA 201 VG (Seppic, Shanghai, China)-adjuvanted rRABV G. One group of mice was immunized with a commercially available human RABV vaccine (Liaoning Chengda, Shengyang, China) using 1/6th of an average human dose per mouse. Phosphate buffer saline (PBS) was used to inoculate control mice. Mice belonging to all the groups received a booster dose of their respective formulation on day 14 post immunization, blood samples were collected on day 14 and 28 post immunization as indicated in Figure 5A.

### Pigs immunization with purified rRABV G

All pigs were maintained and euthanized as per the protocol, approved by the Animal Care and Use Committee (ACUC) of Southern Medical University. Pigs were vaccinated intramuscularly on both sides of neck with either purified rRABV G protein (diluted in 50mM Trehalose, 100 μg per dose) or PBS mixed with aluminium adjuvant three times, for each group, 4 pigs were used. Blood samples were collected at weeks 0, 3, 5, 7 as indicated in Figure 7A, serum IgG binding and neutralizing antibodies were analyzed as mentioned above.

### Statistical analysis

All data were presented as the mean ± SD. SPSS 20.0 software was used for data analysis. The differences in mean values of the positive rate between different groups were analyzed by one-way ANONA test. *p* < 0.05 was considered to be statistically significant.

Correlation analysis between the serum FAVN titers and OD values was also performed by SPSS 20.0 software. Positive values indicate a relationship between* x* and *y* variables such that as values for x increases, values for y also increase. The "0 <*r_s_* <1" (r_s_ = rank coefficient) represents a positive linear correlation. If x and y have a strong positive linear correlation, *r_s_* is close to + 1. An *r_s_* value of exactly + 1 indicates a perfect positive fit. A correlation greater than 0.8 is generally described as strong, whereas a correlation less than 0.5 is generally described as weak.

## Results

### Construction of the RABV G lentiviral expressing plasmid

In this study, a recombinant plasmid pLV-eGFP-RABV G encoding a chimeric protein composed of murine Igκ signal peptide, the soluble form of RABV G, and a histidine tag was first constructed (Figure 1A) and verified by restriction enzyme digestion (Figure 1B) and partial sequencing (data not shown). The Igκ signal peptide was used to make the RABV G secretable, and the histidine tag was designed for detection and purification of RABV G.

### Construction of RABV G recombinant cell lines

**Figure 2:**
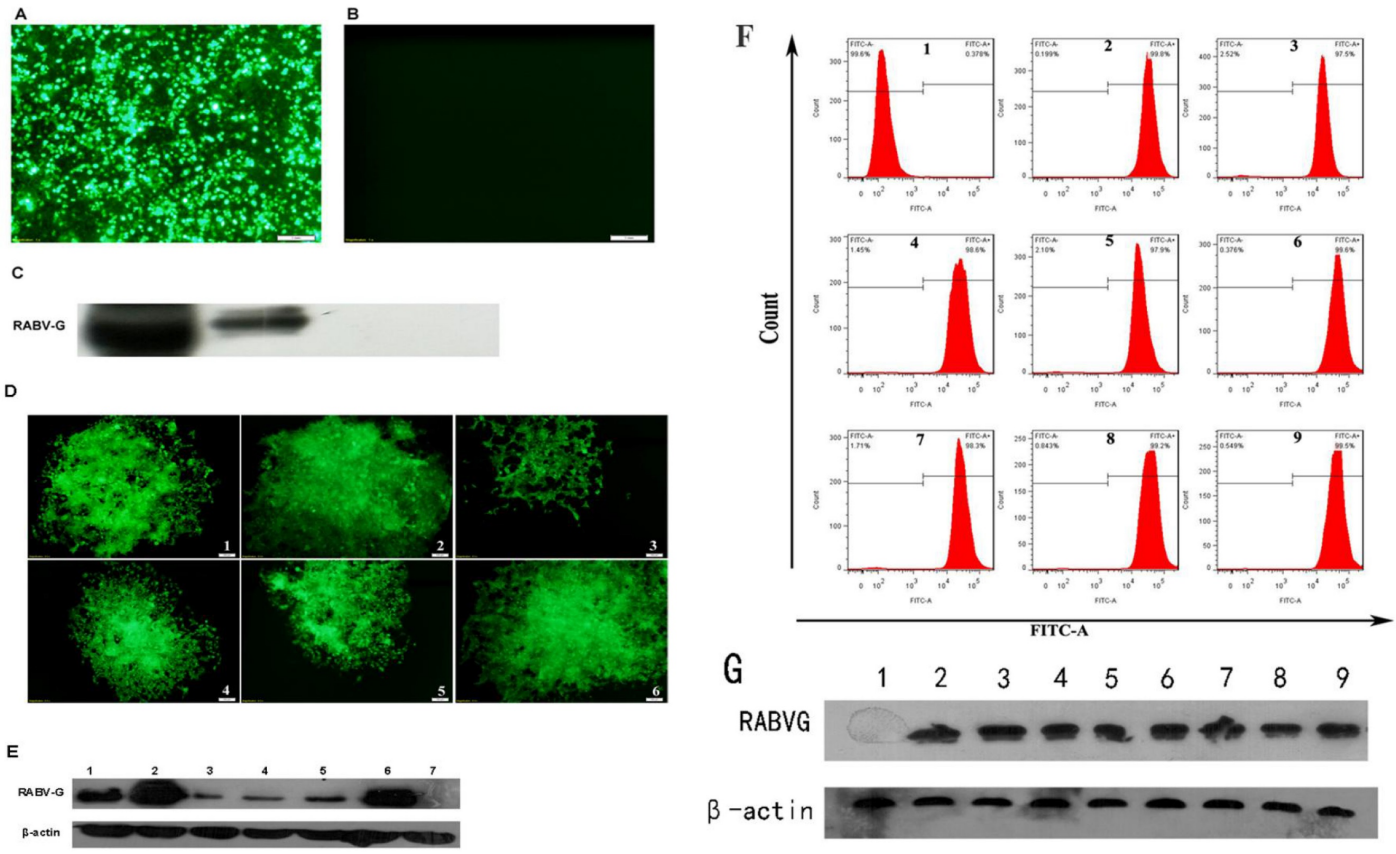
** Construction and identification of recombinant RABV G cell lines.** (A) Fluorescence microscopic image of GFP expression in HEK 293T cells at 48 h post-transfection. (B) Fluorescence microscopic image of untransfected HEK 293T cells. (C) Identification of rRABV G expression. rRABV G was detected by Western Blot using as anti-His antibody at 48 h post-transfection in HEK 293T cells. Lane 1: Cell lysate of HEK 293T cells transfected with pLV-eGFP-RABV G; lane 2: Supernatant of HEK 293T cells transfected with pLV-eGFP-RABV G; lane 3: Cell lysate of HEK 293T cells transfected with pLV-eGFP; lane 4: Supernatant of HEK 293T cells transfected with pLV-eGFP. (D) Fluorescence micrographs of six recombinant HEK 293T cell lines expressing RABV G. (E) The expression levels of rRABV G in six clonal cell lines. Cell culture supernatants were collected and detected by Western Blot using as anti-His antibody. Lane 1 to 6: Cell culture supernatants of clonal cell lines; lane 7: Culture supernatant of HEK 293T cells that transfected with pLV-eGFP. (F) GFP fluorescence ratio and fluorescence intensity of the HEK 293T-RABV G-2. Flow cytometry analysis was performed every five passages. Panel 1: Untransfected HEK 293T cells; panel 2 to 9: HEK 293T-RABV G-2. Cells were collected and analyzed by flow cytometry every five passages. (G) Stability analysis of the clonal HEK 293T-RABV G-2 cells. Lane 1: Culture supernatant of the untransfected HEK 293T cells; lane 2 to 9: Cell culture supernatants of the clonal HEK 293T-RABV G-2. Cell culture supernatants were collected every five passages and rRABV G was detected by Western Blot using as anti-His antibody.

HEK 293T cells were transduced with pLV-eGFP-RABV G. Expression of rRABV G and GFP were determined at 3 days post transduction (Figure 2A & B & C). A total of 6 clonal cell lines for rRABV G were generated by limiting dilution, with the second clone, HEK 293T-RABV G-2, showing highest levels of secreted rRABV G (Figure 2D & E). This recombinant cell line maintained robust and secretory rRABV G expression for at least 40 passages. Western Blot detection of culture supernatants indicated that HEK 293T-RABV G-2 cells exhibited stably secretory expression of rRABV G ectodomain with the size of the approximately 60-kDa. Flow cytometry analysis showed no significant changes in GFP fluorescence ratio and fluorescence intensity (Figure 2F & G).

### Purification and identification of rRABV G

**Figure 3:**
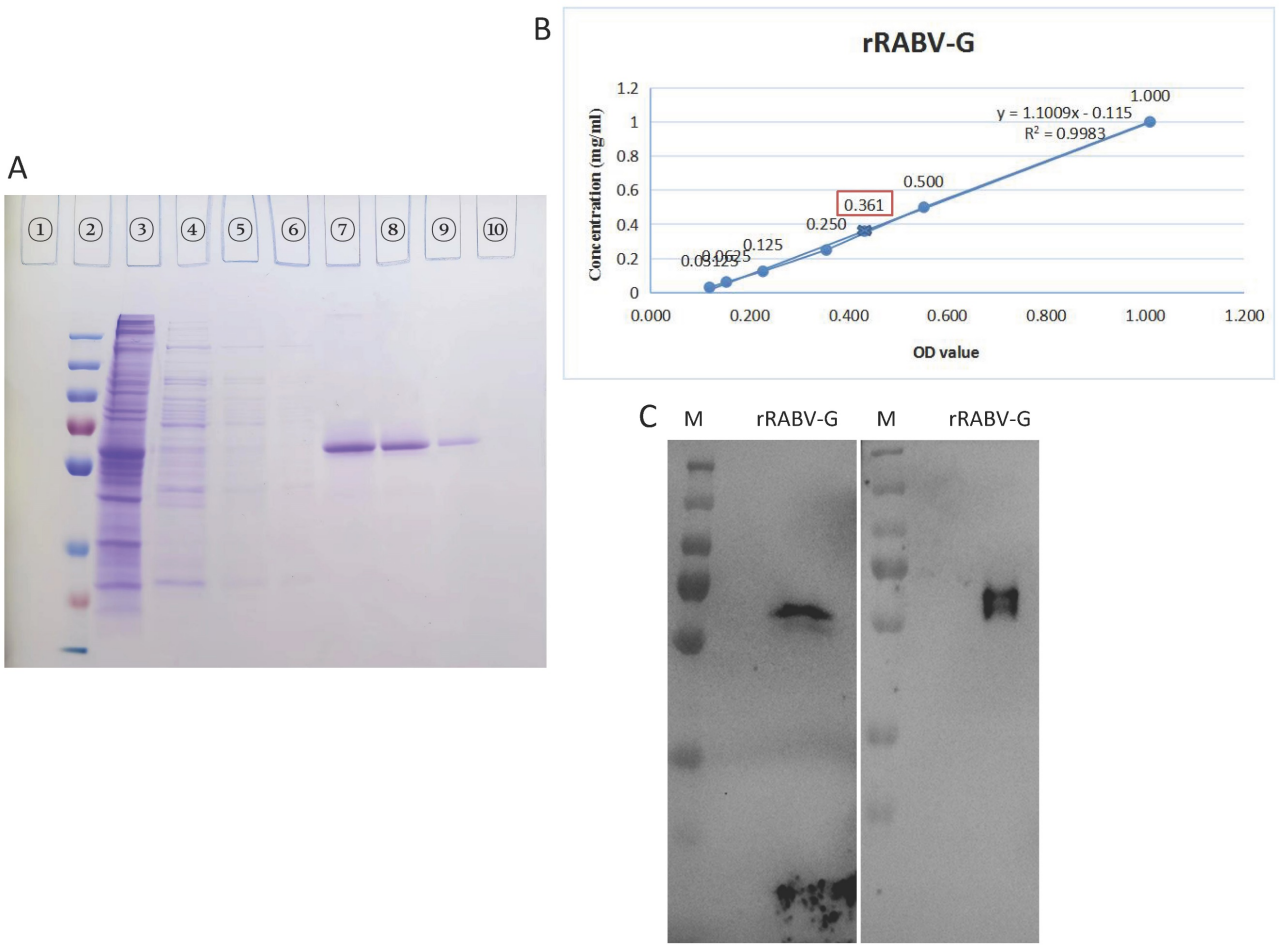
** The purification and identification of rRABV G.** (A) Culture supernatants collected from HEK 293T-RABV G-2 cells were purified by the BioLogic LP protein purification system using nickel affinity columns. Protein purification was analysed by SDS-PAGE. Lane 1: blank; lane 2: protein marker; lane 3: crude culture supernatant; lane 4: flow through from the nickel affinity column; lane 5-6: washing buffer; lane 7-9: elution (500 mM imidazole). (B) Concentration of purified rRABV G detected using BCA Protein Assay Kit. (C) Western Blot analysis of purified rRABV G using the neutralizing anti-RABV G protein mAbs S037 (left) and S053 (right).

rRABV G expressed in the supernatants of stable HEK 293T-RABV G-2 cells was purified using a nickel affinity column. In this experiment, about 200ml supernatant were used. The purified rRABV G was detected by SDS-PAGE and then confirmed by Western Blot. High purity rRABV G was eluted using elution buffer containing 500 mM imidazole (Figure 3A, Lane 7-9), while most of nonspecific proteins were removed with washing buffer containing 100 ~ 200 mM imidazole, with a small amount of rRABV G losing at the same time. The scanned gels were analyzed using Image-Pro Plus 6.0 software and results showed >95% purity. The concentration was 0.361 mg/ml in about 10ml total volume as determined a BCA Protein Assay Kit (Figure. 3B). The purified rRABV G protein samples were further validated by the neutralizing anti-RABV G protein mAbs S037 (Figure 3C, left) and mAbs S053 (Figure 3C, right), results showed specific bands as protein size of ~60 kDa.

### Antigenic property of soluble rRABV G as a diagnostic antigen in ELISA

**Figure 4:**
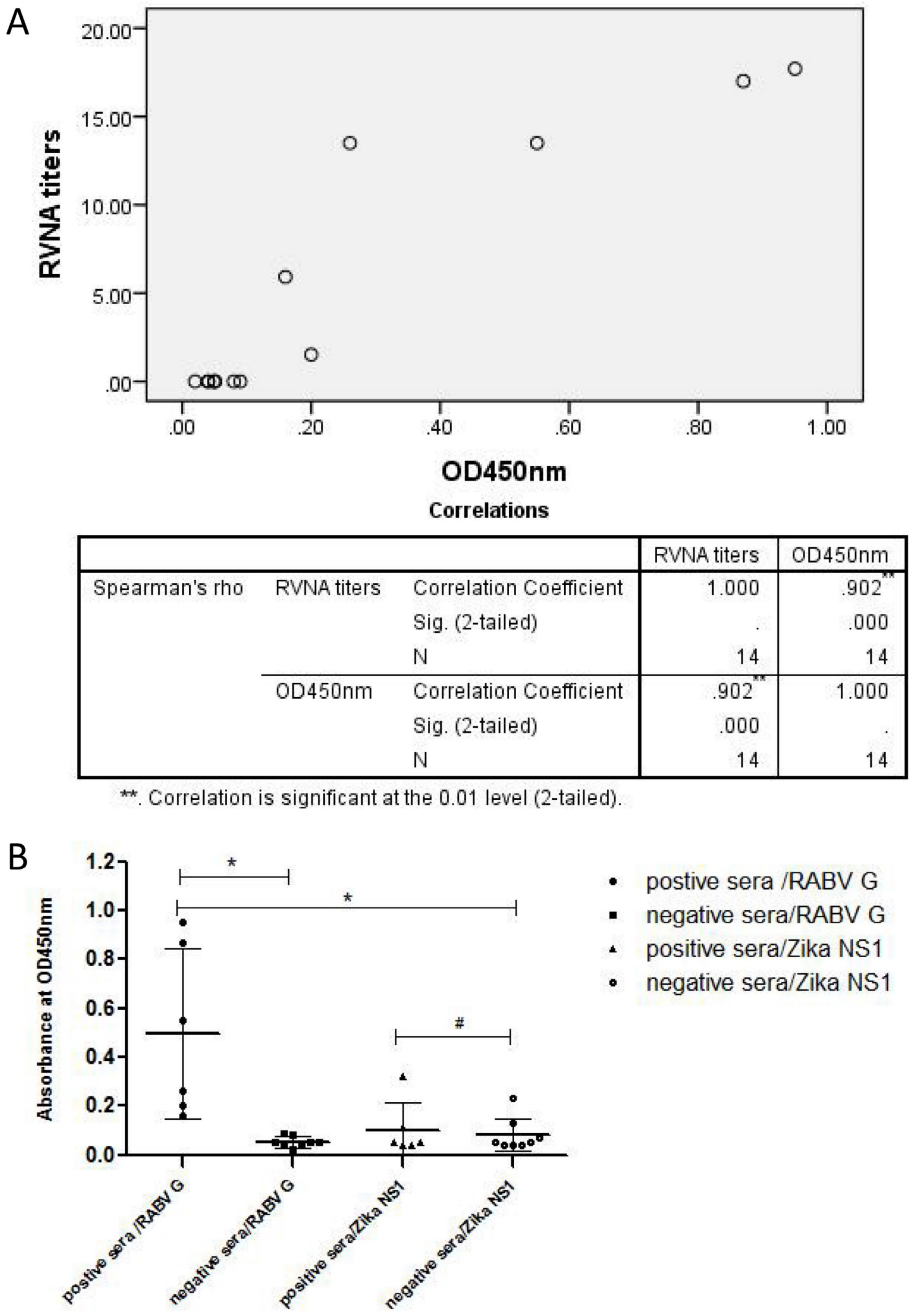
** Antigenic property of rRABV G analyzed by indirect ELISA.** An indirect ELISA was established by using the purified RABV G (100 ng/well) or the control protein Zika virus NS1 as coating antigens. (A) Correlation analysis between FAVN titers and OD values of sera from vaccinated persons were performed by SPSS 20.0 software. A high *r_s_* value (*r_s_* = 0.902, *p* < 0.05) indicated a strong positive linear correlation between FAVN and OD values of these sera. (B) The RABV antibody-positive sera were from persons vaccinated with the human Vero-cell Rabies vaccine while the negative control sera were collected from healthy persons. Statistically significant differences determined using the one-way ANONA test are indicated by asterisks (*:* p* < 0.05 versus the control groups. #: *p* < 0.05 versus the negative serum group).

The purified rRABV G was then used to establish an indirect ELSIA as a coating antigen for detecting anti-Rabies virus antibodies in serum samples from persons vaccinated with human commercial Vero-cell Rabies vaccine manufactured by Liaoning Cheng Da Co., Ltd (PVRV-CD). The levels of neutralizing antibodies against RABV were also measured using fluorescent antibody virus neutralization (FAVN) test. Results showed that there was a strong positive linear correlation (*r_s_* = 0.902, p < 0.05) between the serum neutralizing antibody titers and OD values (Table 1 and Figure 4A). Furthermore, the OD values of all serum samples from RABV-vaccinated persons were greater than the mean + 3SD (0.11) of the OD values of normal human sera (Figure 4B). In addition, there was no significant difference between the OD values of sera from RABV-vaccinated persons and normal human sera when Zika virus NS1 expressed from HEK 293T cells was used as a coating antigen (Figure 4B).

### Immunogenicity analysis of rRABV G in mice

**Figure 5:**
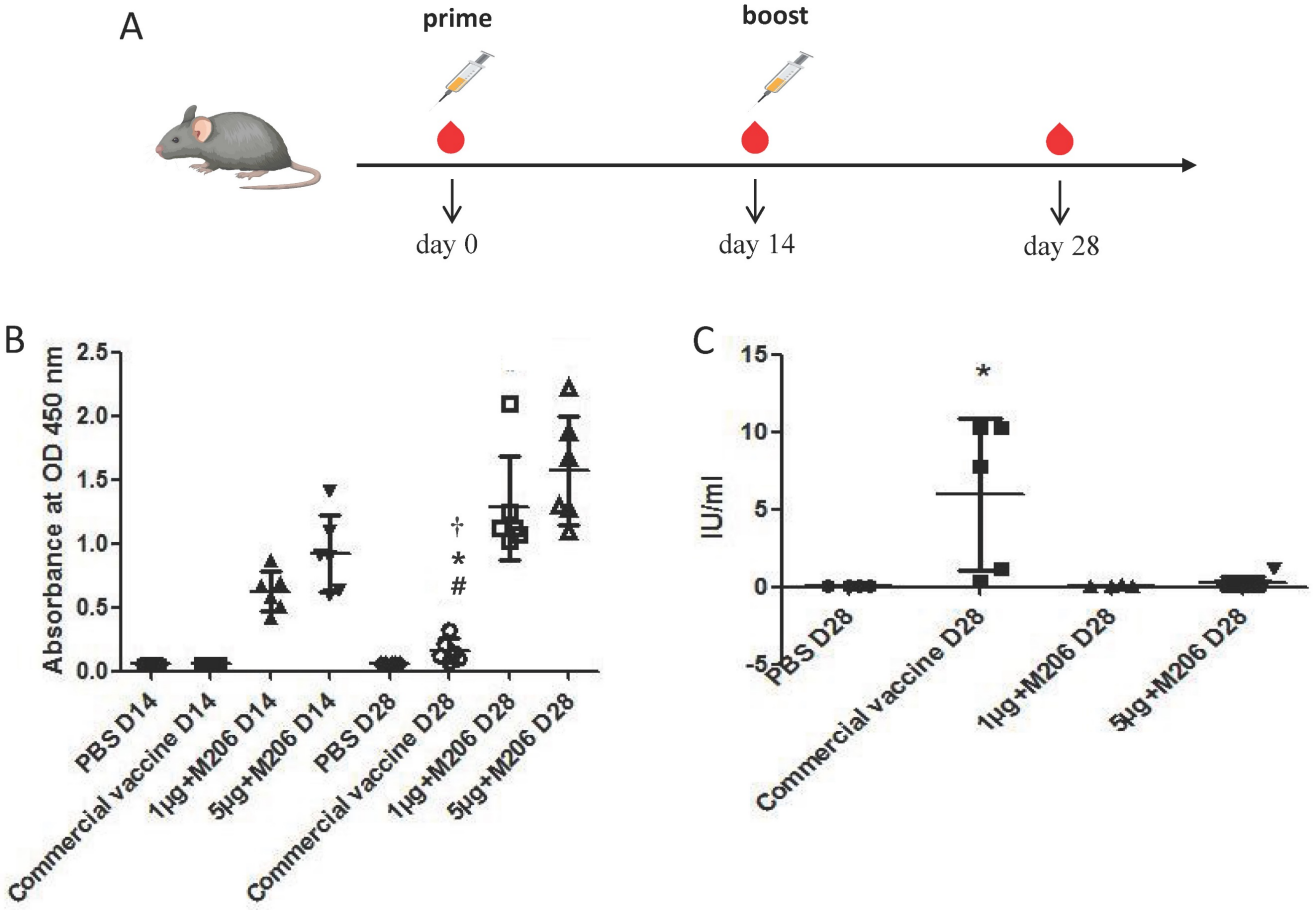
** Immunogenicity analysis of rRABV G in mice.** (A) Blood samples were collected on day 0, 14 and 28 post immunization. (B) Serum antibody responses were measured by ELISA. (C) RVNA titers were measured by fluorescent antibody virus neutralization test (FAVN) in serum samples collected on 28 days post immunization. Statistically significant differences determined using the one-way ANONA test are indicated by asterisks (#: *p* < 0.05 versus the control group PBS on day 28; *: *p* < 0.05 versus the group 1 μg + M201 D14; †: *p* < 0.05 versus the group 5 μg + M201 D14).

To evaluate the immunogenicity of truncated rRABV G, mice were immunized with the purified rRABV G and the specific antibodies against RABV G and RABV neutralizing antibodies were detected in mouse sera by both indirect ELISA and fluorescent antibody virus neutralization (FAVN) test. All animals developed antibodies on day 14 after a single immunization with varying doses of rRABV G. These antibody titers were significantly boosted after a second vaccination (*p* = 0.0288 and *p* = 0.0232 for the 1 μg and 5 μg doses, respectively) (Figure 5B). On the contrary, the commercial human RABV vaccine elicited much lower levels of antibodies against RABV G on day 28 post immunization when compared with purified rRABV G. Also, only seroconversion of RVNA was observed in mice immunized with the commercial RABV vaccine on day 28 post immunization while mice immunized with truncated rRABV G failed to elicit any RVNA titers (Figure 5C).

### Effect of glycine or trehalose on rRABV-G stability during storage

**Figure 6:**
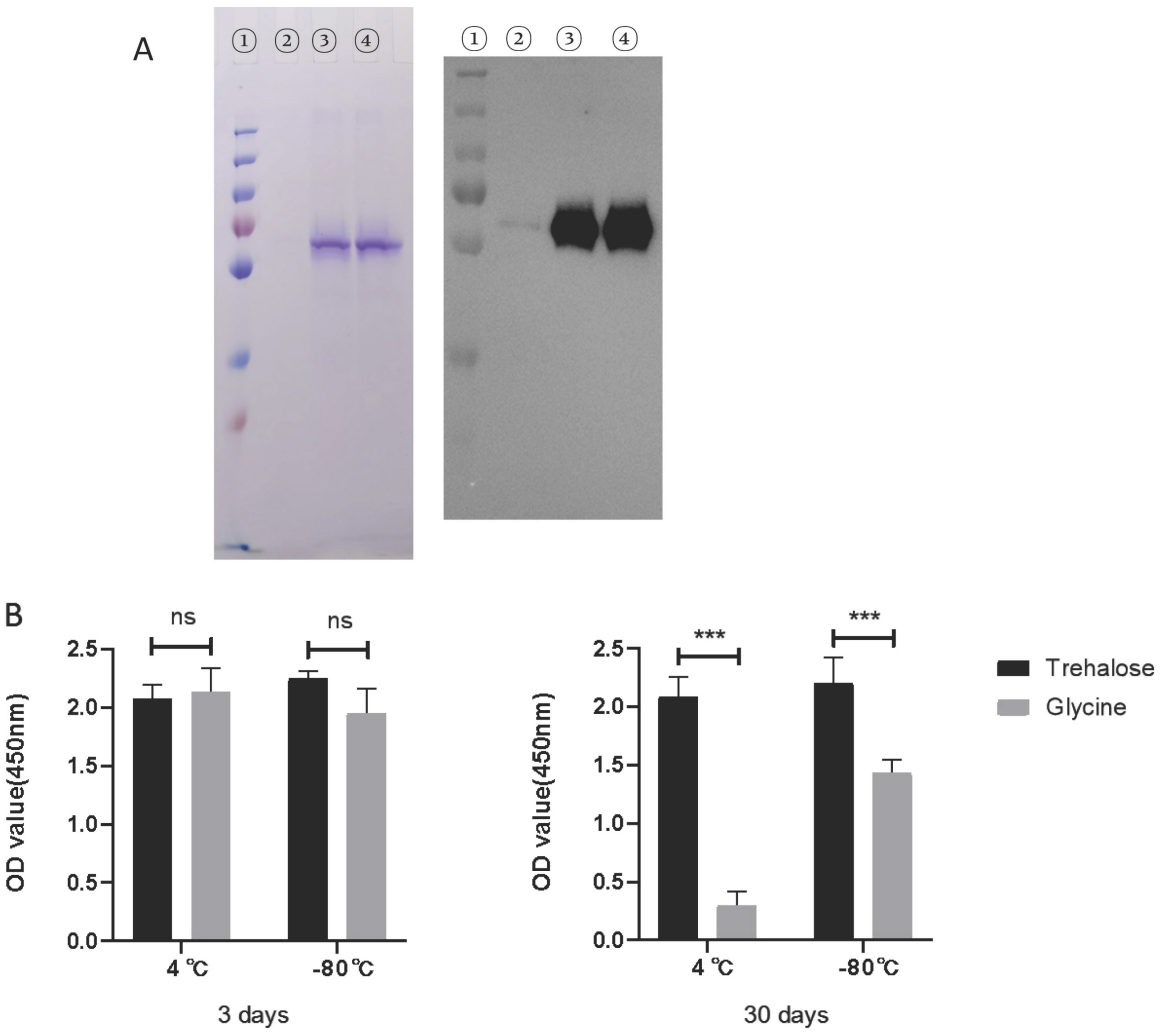
** Stability analysis of rRABV G protein.** The rRABV G protein was diluted in PBS buffer to achieve a final concentration of 100 μg/ml, w or w/o 50mM Glycine or Trehalose. (A) Protein integrity was determined by SDS-PAGE/Western Blot. Lane 1: protein marker; lane 2: rRABV G protein alone; lane 3: rRABV G protein in 50mM Glycine; lane 4: rRABV G protein in 50mM Trehalose. (B) The immunogenicity of rRABV G was determined by indirect ELISA, OD values were performed by SPSS 20.0 software. Statistically significant differences determined using the Unpaired t test are indicated by asterisks (*: p < 0.05).

Based on the findings mentioned above, we initially examined the stability of rRABV-G during storage in a mice experiment using SDS-PAGE and ELISA. The results demonstrated that the rRABV-G experienced significant degradation when stored at 4 ℃ for a week (Figure 6A). This degradation may have been the primary reason for the negative outcomes observed in the mice experiment. To address this issue, we evaluated the protective effects of two different solutions, glycine and trehalose, on the stability of rRABV-G. The results, shown in Figure 6B, confirmed that trehalose alone, at a concentration of 50mM, offered effective protection during both short-term (3 days) and long-term (30 days) storage at both 4℃ and -80℃. These findings suggest that trehalose may serve as a promising protective agent for maintaining the stability of rRABV-G.

### Immunogenicity of rRABV G in pigs

**Figure 7:**
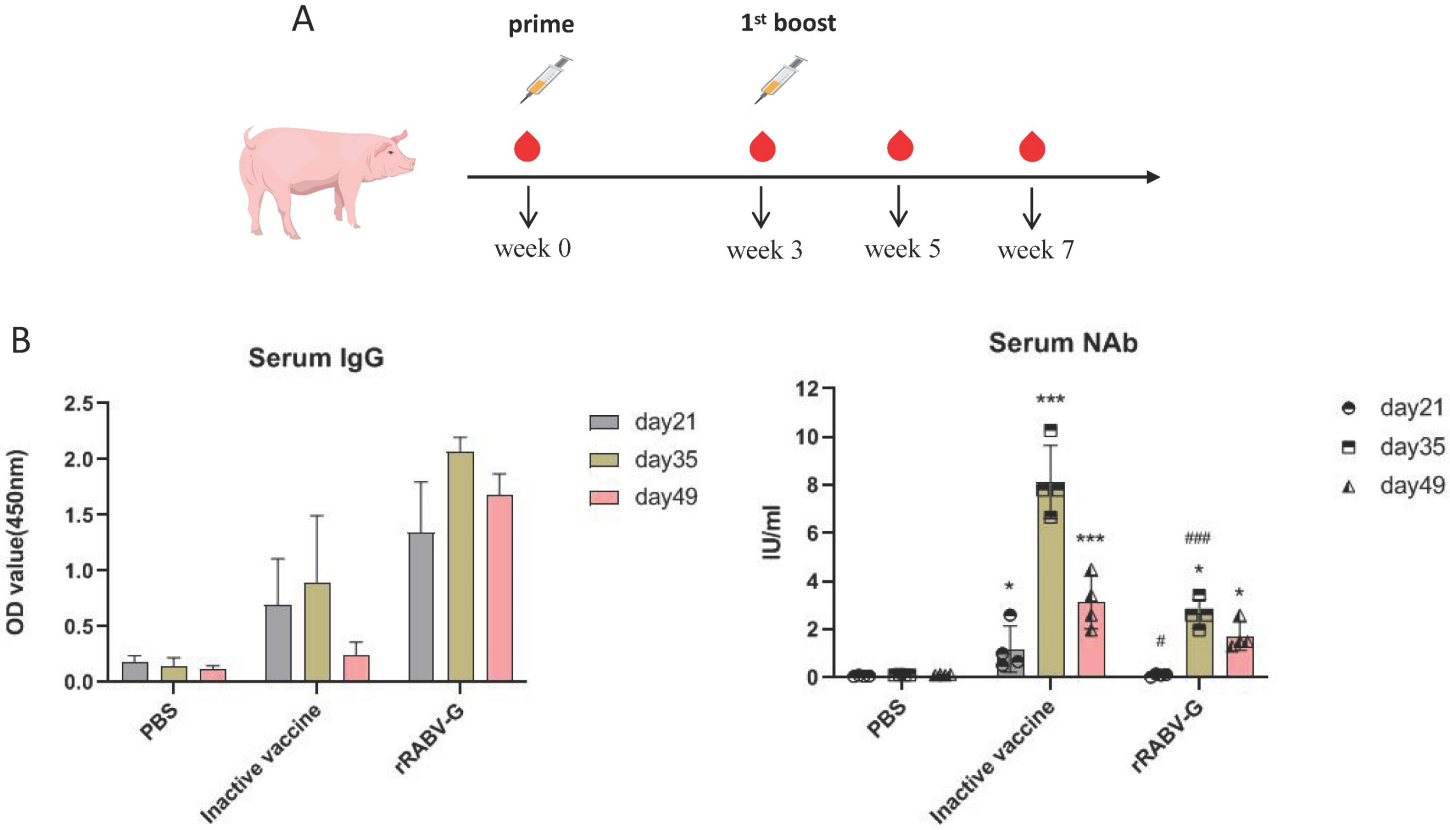
** Immunogenicity analysis of rRABV G in pigs.** (A) Pigs were immunized with 100μg/dose as indicated and blood samples were collected on day 0, 21, 35 and 49 post immunization. (B) Serum antibody responses were measured by ELISA. (C) RVNA titers were measured by fluorescent antibody virus neutralization test (FAVN). Statistically significant differences determined using the one-way ANONA test are indicated by asterisks (#: *p* < 0.05 versus the inactive vaccine group; *: *p* < 0.05 versus the control group PBS).

To further evaluate the immunogenicity of rRABV-G, pigs were vaccinated with two doses of 100 μg purified rRABV-G (protected in 50mM trehalose), pigs vaccinated with commercial inactive vaccine (PVRV-CD) were regarded as positive group. Serum samples collected at different time points post vaccination were used for ELISA and neutralizing antibody detection. The results showed that all vaccinated pigs exhibited a high level of serum IgG antibodies at day 21 after the first immunization (Figure 7B and 7C). However, only those vaccinated with the inactive vaccine showed effective neutralizing antibodies (≥0.5 IU/ml), suggesting that a single dose immunization of rRABV-G was insufficient to induce effective protection in pigs. Therefore, a booster immunization was conducted. The results showed that pigs in the inactive vaccine and rRABV-G groups exhibited a significant enhancement of serum neutralizing antibodies at 2 weeks after the booster, and this level was maintained for at least 2 weeks (Figure 7C). These data suggest that a two-dose immunization is critical for rRABV-G to achieve effective protection.

## Discussion

In this study, we reported the establishment of recombinant cell lines with stable expression of truncated RABV G, the main antigen of rabies virus, using a lentiviral vector. The high purity (95%) rRABV G was obtained using continuous concentration of imidazole and its antigenic property was identified by Western Blot analysis and ELISA using human sera from persons vaccinated with human commercial Purified Vero Cells Rabies Vaccine. However, the truncated rRABV G could not induce RVNAs in immunized mice, although high level of specific antibodies against RABV G has been detected.

Rabies virus G protein is the main structural protein that is exposed and directly involved with viral replication. It is a vital neutralizing antigen and highly conserved. It is a unique antigen that can completely resist rabies and included in all new proposed rabies vaccines 19, 20. After appropriate folding and glycosylation 21, the RABV G becomes immunogenic and can elicit humoral and cell-mediated immune responses 22-24. The RABV G, expressed either as a monomer or in its native trimeric configuration in insect cells, can induce RVNA antibody while the transmembrane-deleted RVBV-G may not have the same immunogenicity 25. Recently, Galvez-Romero et al demonstrated that the linear G5 epitope of RABV G is a potential candidate DNA vaccine development against rabies and the addition of the C3d-P28 adjuvant enhanced protection 26. In the present study, we tried to enhance the immune response against rRBAV-G in mice by using the adjuvant Montanide™ ISA 201. However, the mice immunized with the truncated rRABV G failed to elicit appreciable RVNA although the specific antibodies against RABV G in these mice were much higher than in the commercial human RABV vaccine-immunized mice. Most likely, the rRABV-G protein degraded during storage. It is widely known that the recombinant RABV-G protein is highly unstable in vitro. In this study, we observed that rRABV-G protein underwent significant degradation when stored at 4℃ for just one week. This degradation poses a major challenge in the research and development of rabies virus subunit vaccines. To address this issue, we explored various potential solutions and discovered that the addition of 50mM trehalose served as an effective protectant for the rRABV-G protein (Figure 6). The immunogenicity of the rRABV-G protein protected with the addition of trehalose was further evaluated in pigs. Pigs, being a large genetically outbred animal model, have been widely acknowledged as a reliable model for studying vaccines intended for use in humans due to their highly similar physiologies and immune systems. Recently, the pig model has been utilized to test various vaccine candidates against SARS-CoV-2. In our study, we observed effective neutralizing antibodies (NAb) in pigs immunized with rRABV-G, albeit at a lower level compared to the commercial vaccine group. We thought the cause might be that the candidate recombinant subunit rabies vaccines here was expressed as a monomer, while the native trimeric form of the RABV-G protein was proved to be superior both in terms of immunogenicity and efficacy 27. This discrepancy in NAb levels could also be attributed to differences in antigen dosage and adjuvants used between the two groups.

RABV G in its soluble form has been successfully expressed in many cell systems including prokaryotic cells and eukaryotic cells (yeast, plant, mammal and insect). However, obtaining high levels of rRABV G expression with high purity and stability has been a challenge due to it being unstable and hydrophobic making it hard to purify. The histidine tag-IMAC strategy has been used to purify rRABV G without its transmembrane domain and in denaturing conditions 28. In both cases, the rRABV G may not have immunogenicity, which is consistent with our results. We also found that the purity of the rRABV G was very poor and easily degraded when using a nickel affinity column under standard conditions. We were finally able to obtain high purity (95%) rRABV G by using continuous concentration of imidazole for elution (Figure 3A). Furthermore, there was a positive linear correlation between the serum neutralizing antibody titers and OD values (Table 1 and Figure 4) when the purified rRABV G was used to establish an indirect ELSIA for detecting anti-Rabies virus antibodies in serum samples from vaccinated persons. This suggests that the truncated rRABV G's has potential value as a diagnosis tool. In conclusion, we have generated a recombinant cell line, HEK 293T-RABV G-2, capable of stable and secretory expression of truncated rRABV G. We then established a suitable nickel affinity chromatography purification method for rRABV G. The rRABV G antigenic property was confirmed by Western Blot analysis and ELISA.

In conclusion, this study has successfully developed a simplified and reliable production strategy for recombinant RABV-G protein, consistently achieving high yields of over 50 mg per liter of expression culture. The produced protein can serve as a valuable diagnostic antigen in ELISA and other RABV immunodiagnostic tests. Furthermore, it can also be utilized as a protective antigen in subunit vaccine studies, demonstrating its potential for vaccine development purposes.

## Figures and Tables

**Table 1:** List of RVNA titers (IU/ml) and OD450 values measured by ELISA in human serum samples

Sample	IU/ml	OD450 value	Rabies vaccine inoculation
A*	1.52	0.20	+
B*	17.7	0.95	+
C*	5.92	0.16	+
D*	13.5	0.26	+
E*	17	0.87	+
F*	13.5	0.55	+
1#	/	0.04	-
2#	/	0.08	-
3#	/	0.05	-
4#	/	0.05	-
5#	/	0.05	-
6#	/	0.09	-
7#	/	0.02	-
8#	/	0.04	-

*: Sample A-F: serum samples from persons immunized with human commercial Rabies vaccine. #: Sample 1-8: serum samples from healthy persons who did not undergo Rabies vaccine inoculation.
